# Animals as Communication Partners: Ethics and Challenges in Interspecies Language Research

**DOI:** 10.3390/ani16030375

**Published:** 2026-01-24

**Authors:** Hanna Mamzer, Maria Kuchtar, Waldemar Grzegorzewski

**Affiliations:** 1 Faculty of Sociology, Adam Mickiewicz University, 61-614 Poznań, Poland; mamzer@amu.edu.pl; 2 Faculty of Biology, Nature Conservation and Sustainable Development, University of Rzeszów, 35-310 Rzeszów, Poland; mkuchtar@ur.edu.pl

**Keywords:** interspecies communication, empathy, primates, dogs, research ethics, animal welfare, animal cognition, artificial intelligence

## Abstract

Interspecies communication is increasingly recognized as an affective–cognitive process co-created between humans and animals rather than a one-directional transmission of signals. This review integrates findings from ethology, neuroscience, welfare science, behavioral studies, and posthumanist ethics to examine how emotional expression, communicative intentionality, and relational engagement shape understanding across species. Research on primates, dogs, elephants, and marine mammals demonstrates that empathy, consolation, cooperative signaling, and multimodal perception rely on evolutionarily conserved mechanisms, including mirror systems, affective contagion, and oxytocin-mediated bonding. These biological insights intersect with ethical considerations concerning animal agency, methodological responsibility, and the interpretation of non-human communication. Emerging technological tools—bioacoustics, machine vision, and AI-assisted modeling—offer new opportunities to analyze complex vocal and behavioral patterns, yet they require careful contextualization to avoid anthropocentric misclassification. Synthesizing these perspectives, the review proposes a relational framework in which meaning arises through shared emotional engagement, embodied interaction, and ethically grounded interpretation. This approach highlights the importance of welfare-oriented, minimally invasive methodologies and supports a broader shift toward recognizing animals as communicative partners whose emotional lives contribute to scientific knowledge. This review primarily synthesizes empirical and theoretical research on primates and dogs, complemented by selected examples from elephants and marine mammals, which provide the most developed evidence base for the affective–cognitive and relational mechanisms discussed.

## Simple Summary

Research on interspecies communication—including primates, dogs, and other social species—shows that cognitive and emotional processes in animals are far more complex than traditionally assumed. These findings challenge anthropocentric views in the science of language and cognition. Contemporary ethology, neurobiology, and animal philosophy indicate that communication is not merely the exchange of signals, but a form of participation and relationship. This article integrates biological, veterinary, and humanistic perspectives, analyzing the evolutionary mechanisms of communication, empathy, and morality, as well as the ethical consequences of language research with animals. The authors propose a vision of communication studies in which the human being is no longer the center of inquiry but becomes a partner in shared knowing.

## 1. Introduction

Communication is one of the most fundamental biological functions in any social organism. It enables coordination of behavior, transfer of information, expression of emotions, and maintenance of bonds that are crucial for group survival. In humans, communication takes the form of symbolic language—a system of signs and grammatical rules that allows not only the transmission of content but also the creation of meaning. From a biological standpoint, however, language is only one possible form of communication, not its apex.

For centuries, an anthropocentric view prevailed according to which the capacity for language marked a sharp boundary between humans and other species. Descartes and later modern philosophers sustained the conviction that animals are beings without consciousness, reacting mechanically to stimuli. Charles Darwin challenged this assumption in *The Expression of the Emotions in Man and Animals* [1], arguing that emotional expression has shared evolutionary roots in humans and other animals. In doing so, he initiated a way of thinking about emotions and communication as phenomena that are continuous across species rather than uniquely human.

In the twentieth century, a cognitive turn in the behavioral sciences profoundly reshaped this picture. Ethologists such as Konrad Lorenz and Nikolaas Tinbergen showed that animal communication is not a simple chain of stimuli and responses, but a form of social adaptation that regulates relationships within a group and supports the formation of bonds [2]. In the 1960s and 1970s, the first attempts to teach language to primates launched a far-reaching discussion about the nature of animal cognition and awareness. Experiments by the Kelloggs, the Gardners, Premack, Terrace, and Savage-Rumbaugh [3,4,5,6,7] opened a new conceptual space for reflecting on what language actually is: a tool for transmission, or a way of participating in a relationship.

**Figure 1:**
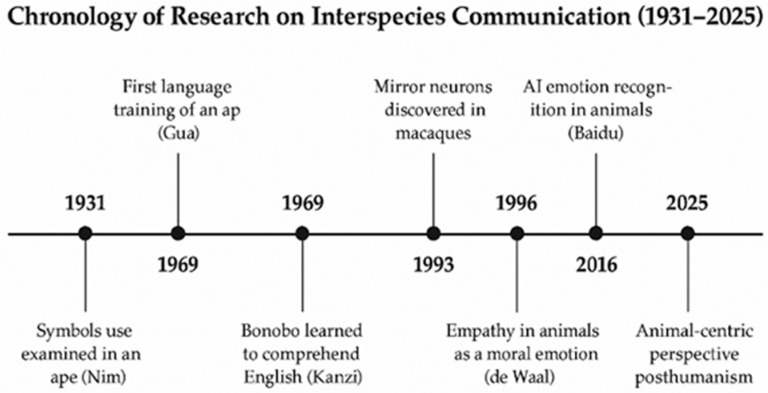
Chronology of key milestones in interspecies communication research (1931–2025). From early ape-human upbringing experiments (Gua; Kellogg & Kellogg [3]) and sign language acquisition (Washoe; Gardner & Gardner [4]) through symbolic communication (Koko; Patterson [11]) and the discovery of mirror neurons (Rizzolatti & Gallese [8]) to recent applications of artificial intelligence in bioacoustics (Earth Species Project and related initiatives [12]. Own elaboration based on literature review.

At the same time, the development of neurobiology provided evidence that the ability to understand others’ emotions and intentions does not require a symbolic language. The discovery of mirror neurons in the premotor cortex of macaques (*Macaca mulatta*) by Giacomo Rizzolatti and Vittorio Gallese revolutionized our understanding of the mechanisms of communication [8]. These neurons fire both when an individual performs an action and when they observe the same action performed by another, forming a neural basis for interpreting intentions and emotions. Neurophysiological studies have shown that mirror systems are closely linked with empathy and prosocial behaviors [9,10]. Figure 1 summarizes the key milestones in the history of interspecies communication research, from early cross-fostering studies to contemporary AI-based projects.

This line of research has been continued and deepened in the work of Frans de Waal, who conceptualizes emotions as the biological language of morality [13,14]. His notion of “evolutionary empathy” links studies of primates, dogs, and other social species with a naturalistic approach to ethics grounded in reciprocity, cooperation, and care. In this framework, communication is redefined as a relational, emotionally saturated process of co-creating meaning rather than a neutral exchange of information. Contemporary animal philosophy and posthumanist theory further challenge the human-centered perspective in which humans are the sole measure of cognition and language [15,16,17]. Donna Haraway’s concept of *companion species* emphasises that our relationships with other animals are co-constructed: we come to know the world *with* them, not merely *about* them. Cary Wolfe similarly calls for an “ethics of participation” that replaces hierarchical, anthropocentric approaches with relational accountability.

From this point of view, interspecies communication research cannot be treated as a purely technical experiment but must be understood as a dialogical encounter. The central question is no longer whether an animal can “speak”, but how it communicates emotions, intentions, and needs—and how humans can learn to interpret and respond to these signals responsibly. The present article therefore aims to present interspecies communication as a multidimensional phenomenon—biological, psychological, philosophical, and ethical. In terms of empirical scope, the review focuses on primate and canine research, with selected references to elephants and marine mammals, as these taxa offer the clearest comparative insights into affective, cognitive, and relational dimensions of interspecies communication. We examine the neurobiological and emotional mechanisms that underpin communication in primates and dogs, outline the moral implications of language and cognition research in animals, and discuss the potential and limits of new technologies, including artificial intelligence, in analysing animal vocalisations and affective states.

Communication—understood not as mere transmission but as shared participation—emerges here as a key domain of contemporary scientific and ethical reflection. As our understanding of animal emotion and intelligence grows, the boundary between “human” and “other” becomes less obvious and more open to critical revision.

## 2. Biological and Cognitive Foundations of Interspecies Communication

Communication in the animal kingdom is a multilayered and cognitively rich phenomenon. It involves not only the transfer of information but also the sharing of emotional states, intentions, and social motivations. Biologically, communication enhances an organism’s fitness by coordinating behavior within groups; cognitively, it allows individuals to infer the internal states of others. Classic ethological works by Tinbergen [2] and Dawkins [18] demonstrated that communicative signals serve both informational and regulatory functions, shaping the dynamics of social interactions.

Contemporary neurobiology, comparative cognition, and behavioral sciences show that communication is deeply intertwined with emotion and emotion—a capacity to detect and respond to another individual’s affective state. In this sense, communication forms the biological foundation for cooperation, bonding, and the emergence of social cohesion.

### 2.1. Neurobiological Foundations of Communication and Empathy

The discovery of mirror neurons in the premotor cortex of macaques (*Macaca mulatta*) by Rizzolatti and Gallese [8] transformed our understanding of communication. These neurons activate both when an individual performs an action and when observing another individual performing the same action, creating a neural substrate for inferring intentions and emotions. Neurophysiological studies demonstrate that mirror neuron activity correlates with empathy, affiliative responses, and emotional contagion [9,10]. The limbic system—including the amygdala, anterior cingulate cortex, and insula—supports this capacity by enabling rapid emotional resonance across individuals. Frans de Waal’s research [13] suggests that empathic behavior in primates and dogs is not exclusively learned but rooted in evolutionarily ancient emotional mechanisms that promote group cohesion. Empathy thus functions as a communicative tool: it enables animals to recognize another’s emotional state and respond appropriately. Emotional contagion has been documented in macaques, chimpanzees and also in dogs reacting to human crying [10,19], pointing to the cross-species continuity of emotional communication.

### 2.2. Evolution and Functions of Social Communication

From an evolutionary perspective, communication increases a species’ ability to survive and reproduce. While rudimentary signaling systems evolved early in invertebrates, sophisticated emotional communication reached its peak in social mammals. Tinbergen [2] classified communicative signals as adaptive behavioral responses, whereas Dawkins and Krebs [18] argued that communication can serve both cooperative and manipulative functions within an evolutionary game.

In primates such as *Pan troglodytes* and *Pan paniscus*, gestures, facial expressions, and vocalizations serve dual roles—emotional and social—supporting bonding, mediating conflicts, and signaling intentions [20]. In many species, vocalizations are tightly integrated with facial and bodily expressions, forming a multimodal “language of emotion”. Studies on dolphins, elephants, and corvids show that complex vocal systems allow individuals to encode identity, status, and motivational states [21,22,23]. Communication thus becomes both a cognitive and social strategy enabling flexible behavioral coordination.

### 2.3. Channels of Communication: From Signal to Emotion

Interspecies communication relies on multiple sensory modalities that interact to create meaningful exchanges. Four principal channels are recognised.

#### 2.3.1. Vocal Channel

Comparative bioacoustic studies show that emotional modulation of vocal signals follows partially universal acoustic rules across species—such as increased fundamental frequency under arousal or greater amplitude variability during negative emotional states—suggesting the presence of a cross-species affective vocal code [19]. In dogs, recognition of human emotions relies on multimodal integration: they process vocal tone, facial expression, and body posture simultaneously, indicating a multimodal rather than linear model of affective perception [24].

#### 2.3.2. Visual Channel

This includes gaze, gestures, posture, facial expressions, and tail movements. Mutual gaze between dogs and humans triggers a bidirectional release of oxytocin, strengthening social bonding and facilitating emotional attunement [25,26].

#### 2.3.3. Chemical Channel

This involves pheromones and chemosignals that convey emotional or physiological states in cats and dogs [27,28].

#### 2.3.4. Tactile Channel

This includes affiliative behavior such as licking, grooming, hugging, and gentle physical contact—all of which reduce stress and reinforce social cohesion [29,30,31].

Cross-species studies show that identical signals may convey different meanings depending on social context and relationship history. Intentionality is central: primates and dogs interpret signals based on situational cues, requiring advanced socio-cognitive processing [32].

Recent comparative studies further emphasise that vocal communication is embedded within broader affective and cognitive systems shared across species. Emotional valence and arousal systematically modulate acoustic features such as pitch, amplitude variability, and spectral noise, creating a partially universal code of affective vocal expression [19,33,34]. AI-driven analyses reveal that vocal structures in corvids, elephants, and marine mammals are shaped by group composition and social context, indicating that emotional communication is both biologically grounded and socially constructed [22,23,34,35]. These findings reinforce that communication signals cannot be interpreted in isolation but require multimodal and relational understanding across species [32].

Additional modalities inaccessible to humans play a crucial role in communication among many species, which rely on substrate-borne vibrations, magnetic-field sensitivity, or echolocation to obtain spatial, social, and emotional information [19,22,34,35,36].

### 2.4. Emotions as a Universal Biological Language

Emotions constitute a fundamental biological language that predates symbolic communication. Across social species, emotional expressions signal internal states and influence the behavior of others. Darwin [1] proposed that emotional expressions share deep evolutionary homologies across species. Current research confirms that many animals identify emotions in others through vocal tone, facial expressions, posture, and olfactory cues. Dogs discriminate emotional expressions on human faces [24], while dolphins modify their vocalizations in response to conspecifics experiencing stress or excitement [35]. Emotions also serve a social role—supporting group cohesion, facilitating reconciliation, and coordinating cooperative interactions. Bekoff [37] emphasizes that emotions function as a “moral language”, where shared affective experiences form the basis for prosociality and fairness.

### 2.5. Empathy, Cooperation, and Social Cognition

Empathy—understood as the capacity to attune to another’s emotional state—is a phylogenetically ancient mechanism supporting cooperation. De Waal argues that empathy evolved to stabilise group dynamics and reduce conflict [14]. Bonobos frequently console distressed individuals through touching, hugging, or sharing food [20]. Dogs adjust behavior to human emotional cues [10,19], and mutual gaze between humans and dogs increases oxytocin levels in both species [26].

In this review, “emotional contagion” refers to rapid affective state matching, whereas “empathy” denotes a broader capacity for affective alignment that may support affiliative or helping responses. “Relational attunement” describes sustained, context-sensitive coordination of affect, attention, and action.

These processes are supported by deeply conserved affective systems involving subcortical and limbic circuits, including amygdala-centered salience processing and hypothalamic–brainstem pathways [38].

Empathy and cooperation thus form the emotional–cognitive foundation of communication. In primates and dogs, emotional signalling is integrated with social cognition, creating what may be described as an “emotional language”—a relational system based on shared affect and mutual responsiveness.

Additional evidence shows that empathy and cooperation arise from deeply conserved affective systems. Emotional contagion, shared arousal, and affiliative responses correspond with coordinated limbic activation across species [10,39,40]. Comparative welfare science demonstrates that positive emotions promote approach behavior, social bonding, and cooperative strategies in domesticated and wild animals [41,42]. These mechanisms interact with social cognition: animals interpret emotional cues not merely as signals but as invitations to relational engagement, forming stable cooperative bonds [37,43].

### 2.6. Summary

The biological and cognitive foundations of interspecies communication demonstrate that the capacity to share meaning, emotion, and intention is not unique to humans. Evolutionary continuity of emotional, neural, and social mechanisms indicates that communication is a shared heritage across many species.

Research on primates and dogs reveals that communication extends far beyond information transfer—it encompasses empathy, cooperation, and relational awareness. Understanding communication as a relational process highlights the need for ethical, respectful interactions with non-human animals and sets the stage for further analysis of primates and dogs as communicative partners in the sections that follow.

## 3. Primates as Communicative Partners

### 3.1. Primates in Language and Cognition Research

Research on communication with non-human primates forms a central chapter in the study of animal cognition. Early cross-fostering experiments by the Kelloggs showed that primates can understand human gestures and words but lack the anatomical capacity for speech [3]. The Gardners demonstrated that chimpanzees can acquire and transmit signs in American Sign Language [3]. Premack’s symbolic-language experiments revealed that primates can use abstract categories rather than relying solely on conditioned associations [5]. Terrace’s critique of Nim Chimpsky reframed the debate by distinguishing between imitation and genuinely generative communication [6].

Subsequent analyses—including Patterson’s work on gorilla pragmatics and studies of gesture organisation—show that primate communication exhibits intentional, context-sensitive patterning rather than mechanistic repetition [11,44,45]. Comparative ethics further indicate that language-trained primates experience emotional dependency and distress when deprived of adequate social interaction, underscoring the need for methodological reform in cognitive research [46,47,48]. These insights shift the focus from symbolic competence to the emotional and relational conditions under which communicative behavior acquires meaning.

### 3.2. Emotional and Symbolic Expression in Primates

Koko and Chantek demonstrated that apes can use signs to express emotions, humour, preferences, grief, and autobiographical information [11,44]. These findings challenged earlier assumptions that primate communication is limited to instrumental gestures. Emotional expression in apes proved complex, context-dependent, and relational.

Recent neurocognitive models propose that ape emotional expressivity reflects integrated affective–cognitive processing rather than simple behavioral output [39,40,49]. Observations of consolation, empathy-driven reunification, and post-conflict repair show that primates use gestures and vocalisations to regulate social tension and restore affiliative bonds [20,45]. Welfare studies document that relational deprivation leads to declines in mood and motivation, reinforcing the ethical imperative to treat apes as communicative subjects with complex emotional needs [41,47,48].

### 3.3. Consciousness and Theory of Mind

Mirror self-recognition in chimpanzees and orangutans provides evidence of self-awareness [50]. Theory-of-mind abilities include attributing knowledge, intentions, and emotional states to others [39,51]. Bonobos and chimpanzees use gaze, gesture, and touch to monitor the intentions and attentional states of their social partners.

Further research suggests that theory-of-mind capacities vary with social structure, developmental exposure, and opportunities for cooperative interaction [45,51,52]. Behavioral and neurobiological studies show that primates flexibly adjust attention, gesture use, and gaze direction depending on partner knowledge and emotional state, indicating dynamic, relational forms of mental-state attribution rather than fixed behavioral rules [39,49].

### 3.4. Symbolic Language and Spontaneous Learning: The Case of Kanzi

Sue Savage-Rumbaugh’s studies with bonobos revolutionized the field. Kanzi, raised in a human-centered environment, learned to use lexigrams—graphic symbols representing concepts—without formal instruction [7]. Unlike earlier subjects, Kanzi acquired symbolic communication through observation.

Kanzi produced multi-symbol utterances, responded accurately to complex commands, and demonstrated comprehension of both semantics and pragmatics. When asked to “put the soap in the refrigerator” he performed the action correctly—showing understanding beyond simple stimulus–response patterns.

Kanzi also reacted emotionally to the tone and affect of researchers, and his behavior revealed sensitivity to the emotional context of interactions. This body of work initiated the field of relational ethology, interpreting communication as a *shared experience* rather than a mechanical exchange.

### 3.5. Emotions, Empathy, and Morality in Primates

Empathy in primates manifests at both emotional and cognitive levels. Research by de Waal [13,14] shows that chimpanzees, bonobos, and macaques console distressed individuals, embrace them, or share food—behaviors rooted in emotional attunement rather than conditioning.

Brosnan and de Waal [53] revealed a sense of fairness in capuchin monkeys: they refused rewards when a partner received a better one for the same task. This demonstrated that moral behavior evolves from emotional mechanisms—*feeling precedes reasoning*.

Ethically, these findings challenge traditional views of animal status. If primates grasp emotions, respond compassionately, and suffer psychological distress, they must be recognized as subjects with moral relevance.

### 3.6. Ethical Consequences of Language Research

Language experiments produced scientific breakthroughs but also exposed emotional suffering in research animals. Individuals such as Nim Chimpsky experienced depression and self-harm after being transferred to laboratory facilities [54]. Modern ethical frameworks, including Directive 2010/63/EU [55], require minimizing distress and providing strong justification for using primates in research. Posthumanist thinkers such as Haraway [15] and Wolfe [16] argue that true ethical practice must move beyond “harm reduction” toward recognizing animals as *co-participants in knowledge*. In this view, the human–animal research relationship becomes a dialogue rather than an extraction of data. The animal is a partner, not a tool.

### 3.7. Communication as a Relational Process and the Fluidity of Species Boundaries

Studies on primates show that communication is not binary but exists along a continuum. Bonobos and chimpanzees transmit information, share emotions, and participate in intersubjective exchanges. Marc Bekoff [37] argues that understanding animals requires “zoomorphizing” the human—adopting the animal’s emotional perspective rather than imposing human categories. Haraway’s concept of *cognitive companionship* [15] positions communication as a co-created process rooted in shared presence and mutual attentiveness. From this viewpoint, the “boundary” between human and non-human becomes porous. Meaning emerges through relationship, not domination.

### 3.8. Summary

Primates demonstrate that language, emotion, empathy, and morality share evolutionary origins. Research on *Pan paniscus*, *Pan troglodytes*, *Gorilla gorilla*, and *Pongo pygmaeus* reveals that communication is more than information transfer—it is a relational, emotional, and ethical phenomenon.

In an interdisciplinary context, interspecies communication emerges as a form of shared cognition. The next section presents a species that co-evolved with humans and developed a unique emotional and cognitive communication system: the domestic dog (*Canis familiaris*). Table 1 summarizes major milestones in interspecies communication and language research, from classical primate studies to AI-enabled approaches.

## 4. Dogs as a Contemporary Model of Communication and Empathy

### 4.1. Introduction—The Dog as an Evolutionary Partner of the Human

The relationship between humans and dogs (*Canis familiaris*) represents a unique convergence of biology, emotion, and culture. The domestication of the dog, initiated approximately 30–40 thousand years ago [57], resulted in a species that not only shares physical space with humans, but also mirrors their emotional lives to a remarkable extent. Dogs are not laboratory animals—they are co-participants in human existence, partners in communication, collaborators, and emotional companions.

Contemporary cognitive and neurobiological studies confirm that the dog is an exceptional model for examining interspecies communication. Its ability to interpret human gestures, emotions, and intentions far exceeds that of other domesticated animals. As noted by Miklósi [25], the dog is “a social species evolutionarily prepared for cooperation with humans”.

### 4.2. Evolution of Cooperation and Communication with Humans

The domestication of the dog was not solely a biological process, but also a cognitive one. Studies supporting the domestication hypothesis [58] indicate that natural and artificial selection favored individuals that were more empathetic, less emotionally reactive, and better able to cooperate. This led to the emergence of a species whose communicative strategies evolved in parallel with those of humans.

Dogs display extraordinary sensitivity to human social cues. They can follow gaze direction, interpret pointing gestures [32], recognize intentions, and detect emotional tone in human vocalizations [59]. Importantly, they also modulate their own behavior according to the emotional state of their human partners. This makes them a natural model for studying interspecies empathy.

### 4.3. Neurobiology and Emotions in Dogs

Advances in neuroimaging—particularly fMRI performed on awake, unrestrained dogs—have enabled the analysis of canine neural responses to human emotional signals. Andics et al. [59] demonstrated that activation of the canine auditory cortex differentiates human vocal emotional tones, and that brain regions involved in emotion processing are homologous to those found in humans. Homologous components include limbic and paralimbic regions involved in affective processing. Canine neuroimaging studies indicate that emotionally salient social contexts recruit neural systems functionally comparable to human affective networks [60,61].

Neurohormonal studies by Nagasawa et al. [26] confirmed that mutual gaze between a dog and a human increases oxytocin levels in both species, forming a biological bonding loop. This is the same mechanism observed in mother–infant attachment. Interaction with a dog not only activates the human reward system but also lowers cortisol levels and reduces heart rate, indicating a measurable physiological impact of emotional bonding.

**Figure 2:**
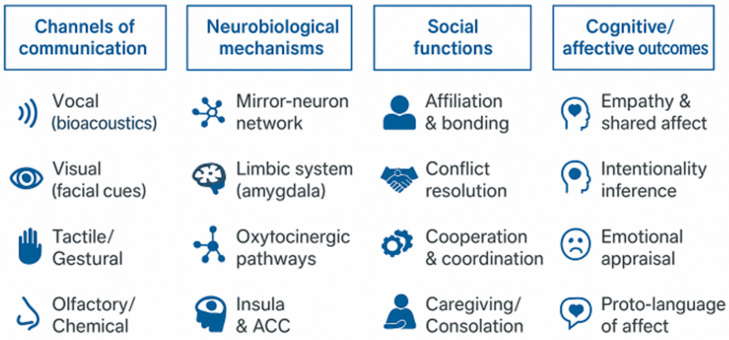
**Evolutionary and neurobiological bases of interspecies communication.** Schematic overview of four integrated layers: Channels (vocal/bioacoustic, visual/facial, tactile/gestural, olfactory/chemical) → Neurobiological mechanisms (mirror neuron network, limbic/amygdala, oxytocinergic pathways, insula & ACC) → Social functions (affiliation, cooperation, conflict resolution, caregiving) → Cognitive/affective outcomes (empathy, intentionality inference, emotional appraisal, proto-language of affect). Own elaboration based on Rizzolatti & Craighero [62]; Gallese [39]; Andics et al. [59]; Nagasawa et al. [26].

Dogs’ empathic responsiveness has also been documented behaviorally. In the classic study by Custance and Mayer [10], dogs spontaneously approached crying individuals, showing affiliative behaviors consistent with emotional rather than conditioned responses. The relationships between communication channels, neurobiological mechanisms, and social functions of emotions are shown in Figure 2.

### 4.4. Social Cognition and Intentionality

Dog–human communication relies not only on stimulus reading, but also on interpreting intentions. As Kaminski notes [63], dogs can distinguish situations in which a human *does not want* to perform an action from those in which a human *cannot* perform it—an ability requiring an understanding of goals rather than mere observation of movement.

This level of social cognition is especially evident in cooperative tasks such as working with handlers, tracking, or responding to verbal and non-verbal commands. Dogs also “read” human attention: they refrain from stealing food when a human is watching, but do so readily when the human is turned away [64]. Such behaviors suggest the presence of a rudimentary “theory of mind”—the capacity to attribute cognitive states to others.

### 4.5. Emotions, Empathy, and Welfare—Behavioral and Ethological Perspectives

From the perspective of modern ethology and applied behavior science, canine emotions are central to welfare. Welfare cannot be defined solely in physiological terms such as physical health, stress levels, or nutrition; it must also include emotional and cognitive dimensions—an animal’s capacity to feel and express emotions, exert environmental control, and maintain social relationships.

Traditional welfare models focused on avoiding pain and suffering have been expanded to include the affective dimension, acknowledging that animals experience both negative and positive emotions [37]. Contemporary research on affective physiology demonstrates that positive experiences—such as social contact, environmental exploration, or play—modulate autonomic activity and neurohormonal markers of welfare, including dopamine and oxytocin levels [26].

Behavioral models integrating neurobiology, affective science, and cognition emphasize that emotions serve informational, motivational, and adaptive functions. As Panksepp argues [40], emotions are primary motivational systems essential for the survival of social species. In dogs, emotions play a central role in learning and communication with humans, shaping empathy and cooperation.

Experimental studies confirm dogs’ empathic responses to human affect. Custance and Mayer [10] showed that dogs spontaneously comfort crying individuals, exhibiting affectively appropriate behaviors. Neuroimaging studies by Andics et al. [59] reveal homologous brain structures for processing emotional prosody in dogs and humans.

Emotional welfare therefore includes physiological markers (heart rate changes, cortisol variation, muscle tension) and cognitive processes (expectation, stimulus interpretation, perceived control). This aligns with the concept of affective cognition, which integrates emotional processes with learning and motivational mechanisms [65].

Ethology increasingly recognizes that emotionality—not intelligence—is the core of dog–human communication. The ability to feel positive and negative emotions, interpret partner intentions, and respond to affective states creates a unique system of interspecies empathy. As Bekoff notes [37], emotions are not only internal states but also mechanisms of social regulation and biological foundations of morality.

According to Mellor’s “Five Domains” model, welfare assessments must consider not only physiological indicators of stress but also positive affective experiences such as comfort, security, and social satisfaction [66]. Neurophysiological research further confirms that positive emotions modulate stimulus perception and learning processes, meaning that affective state directly influences behavioral outcomes [41].

### 4.6. The Emotional Language and Relational Nature of Dog–Human Communication

From an ethical and philosophical perspective, communication between humans and dogs is dialogical. It involves not merely transmitting commands but mutually adjusting behaviors and emotions to create a shared space of meaning. Donna Haraway describes this phenomenon as a *companion species* relationship in which dog and human co-construct a shared world of experience [15].

This relational perspective is supported by research on neurobiology of emotion. According to Panksepp’s affective systems theory [40], dog–human interactions involve shared primary affects—attachment, fear, joy, curiosity—which underlie empathy and bonding. This process, termed *affective synchronization*, involves real-time alignment of emotional and physiological states.

Neurohormonal studies demonstrate that eye contact and touch between humans and dogs activate the oxytocin system in both species, strengthening bonds and enhancing a sense of safety [26]. This mechanism, known as the *oxytocin–gaze positive loop*, is one of the best—documented biological pathways of interspecies emotional communication.

Dogs react to human vocal tone, facial expressions, and gestures, while humans interpret canine behavior in terms of emotion and intention. Andics et al. [59] showed that dog brains differentiate human speech based on emotional prosody, indicating a shared cross-species system of affective perception.

This emotional synchrony results in *affective coherence*—a state in which partners’ emotions become mutually coupled, forming a shared communicative field. This creates what scholars describe as an “emotional language”—a biologically rooted communication system based on tone, rhythm, facial cues, and touch rather than symbolic syntax [13,37].

### 4.7. Dogs in Research on Consciousness and Morality

Canine cognitive and emotional capacities have become central to comparative cognition and social ethology. Studies show that dogs not only understand human gestures and social cues, but also display behaviors indicative of self-awareness, empathy, and proto-morality.

De Waal argues that morality is not exclusively human but emerges from emotional social systems such as compassion, cooperation, and fairness [13,14]. Inequity aversion—well documented in primates—has also been observed in dogs. Brosnan and de Waal demonstrated that capuchin monkeys refuse to cooperate when treated unfairly [53], and analogous responses have been found in dogs: individuals stop performing tasks when they observe conspecifics receiving better rewards for identical actions [43].

Dogs also show sensitivity to human intentionality. Kaminski and Nitzschner reported that dogs distinguish situations in which a human *does not want* vs. *is unable* to perform an action [32], indicating basic first-order theory of mind.

Neuroimaging studies by Andics et al. [59] reveal that dogs process emotional vocal cues in brain regions homologous to those of humans, supporting the presence of shared neural mechanisms of social communication. Behaviorally, dogs respond to human crying with affiliative consolation behaviors [10], and eye contact triggers oxytocin-mediated bonding [26].

Bekoff notes that dogs regulate emotions, adhere to social reciprocity, and recognize distress in others [37]. During play, they use calming signals and modify behavior when partners show discomfort—an indicator of emotional self-regulation and moral sensitivity.

Classic experiments by Range and colleagues showed that dogs stop performing tasks when they observe another dog receiving a superior reward for the same behavior, demonstrating inequity aversion based not merely on conditioning but on emotional social comparison [43].

### 4.8. Ethics of the Relationship and Human Responsibility

The human–dog relationship represents one of the most significant examples of interspecies emotional and moral connection. Ethically, humans bear responsibility for the welfare of a species whose emotionality and awareness are scientifically established. Bekoff argues that dogs possess “the right to emotion”, and that psychological suffering is as real as physical pain [37]. Research on canine behavior must therefore adopt a participatory and respectful approach recognizing dogs as subjects rather than objects.

**Figure 3:**
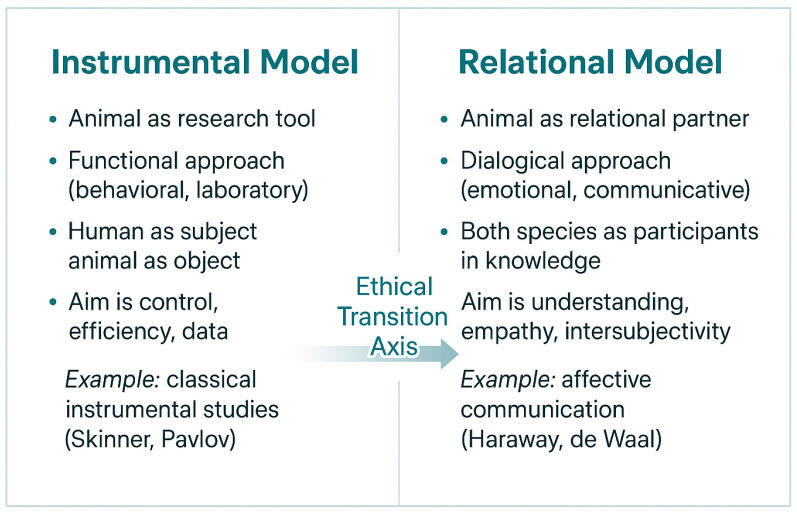
**Ethical models of the human–animal relationship: instrumental versus relational.** The figure contrasts two paradigms: an instrumental model based on hierarchy and utility, and a relational model grounded in empathy, reciprocity, and co-agency. The gradient transition reflects the ethical shift from control to participation and from anthropocentrism to shared moral presence. Own elaboration based on Haraway [15], Wolfe [16], Bekoff [37], de Waal [13]. A comparative overview of ethical theories relevant to interspecies communication is presented in Table 2.

From a posthumanist perspective [15,16], communication between humans and dogs is a shared cognitive experience, not a one-sided process. Dogs and humans co-create meaning, learn from one another, and exchange emotions. This contrast between **instrumental** and **relational** models of human–animal interaction is illustrated in Figure 3.

**Table 2:** **Comparative models of ethics in interspecies communication research.** The table summarizes key ethical frameworks addressing human–animal relations, emphasizing communication, agency, and moral responsibility. It contrasts utilitarian ethics (Singer) [67], feminist posthumanism (Haraway) [15], animal citizenship theory (Kymlicka & Donaldson) [17], and post-structural ethics (Wolfe) [16], highlighting the shift from instrumental to relational ethics.

Ethical Model	Core Principles	View on Communication
**Utilitarianism**	Moral value derived from the capacity to experience pleasure and pain; ethical aim	Communication as a tool for identifying sentience and extending moral concern to non-human animals
**Feminist** **Posthumanism**	Ethics of care and relation responsibility; emphasis on embodied	Communication as reciprocal interaction and co-creation of meaning between species
**Animal Citizenship Theory**	Political and moral co-agency; recognition of animals as members	Communication as participation and mutual recognition within moral and civic relationships
**Post-Structural Ethics**	Deconstruction of species boundaries; focus on discourse, language and representation	Communication as a space of ethical intersubjectivity beyond species categories

In this approach, the researcher–animal relationship is a dialogue, not an experiment. The animal participates in the process of knowledge creation, and humans bear moral responsibility for the consequences of this encounter. The boundary between ‘human’ and ‘another species’ becomes conventional. Communication becomes a shared language of emotions and consciousness, in which meanings are not assigned unilaterally but co-created. As Haraway writes, true ethics begins when we see in the other not an object, but a presence [15].

### 4.9. Summary

As a species that co-evolved with humans, the dog represents an exceptional model for studying communication, emotion, and consciousness. Its capacity for empathy, cooperation, and understanding intentions places it among the cognitively and emotionally closest species to humans. Biological, neurobiological, and ethological research confirms that dog–human communication is not a one-directional transfer of signals, but a dynamic and relational process of emotional co-regulation [13,15].

From the standpoint of research ethics and posthumanist philosophy, this relationship becomes a cognitive dialogue in which humans and animals co-construct meaning. Haraway emphasizes that in interspecies communication, the boundary between observer and subject becomes blurred—each encounter is an exchange of knowledge and emotion [15].

Studies of emotions and empathy in dogs and primates support de Waal’s thesis that cooperation and morality originate from emotional systems rather than purely rational cognition [13,14]. Bekoff similarly argues that emotions are a biological language of coexistence, grounding empathy and social stability [37].

Thus, dog–human communication emerges as a model of relational ethics and co-participation, integrating scientific, philosophical, and welfare perspectives. Emotions and cognition appear as shared evolutionary heritage, and understanding the other—regardless of species—requires empathy and cognitive openness. In this sense, the dog is not merely an “object of research” but a participant in knowledge formation and a co-creator of shared emotional experience.

## 5. Ethics of Research on Interspecies Communication

### 5.1. Introduction—Limits of Knowledge and the Responsibility of Science

Research on interspecies communication, especially in relation to animals with highly developed cognition—such as primates, dolphins, corvids and dogs—requires particularly careful ethical reflection. As scientific knowledge about animal empathy, emotions, and consciousness advances, the question of the limits of experimentation takes on a new meaning.

In the twentieth century, an instrumental perspective dominated: the animal was treated as a “model” used to understand human cognitive processes. However, discoveries of the last decades—from mirror neurons to studies on theory of mind in primates—have made it clear that the animal is a subject capable of experiencing suffering, fear, frustration, but also curiosity and joy [11,40]. This changes the moral position of animals in science and demands a redefinition of the researcher’s role.

Ethics of research on communication cannot be reduced to formal compliance with procedures; it must also encompass reflection on the cognitive relationship that arises between humans and the species under study. The researcher’s responsibility concerns not only the physical welfare of animals, but also their emotional and cognitive well-being [8,37].

As Haraway notes, every encounter between a human and an animal is a moral event—a space of mutual influence and response [15]. This means that scientific knowledge is not a one-sided act, but a form of co-participation in which both parties shape meanings and emotions.

Contemporary research on animal emotions and consciousness, conducted within relational ethology and affective neuroscience, confirms that emotional experience is an integral component of cognition [37,40]. In this sense, every attempt to study interspecies communication requires both empirical rigour and ethical sensitivity—an understanding that observation transforms not only the research object, but also the researcher.

Modern research ethics, particularly in studies involving animals, is moving away from a model of domination towards a relational model—grounded in co-participation, empathy and recognition of animals’ cognitive subjectivity [15,16]. This direction of development forms the foundation of contemporary bioethics, in which understanding becomes a form of responsibility, and knowledge—a moral practice.

### 5.2. Evolution of Ethical Principles in Animal Research

The first ethical codes concerning animal experimentation emerged in the mid-twentieth century and were primarily protective in nature. Documents such as *The Guide for the Care and Use of Laboratory Animals* (National Research Council, 1963) [68] and later recommendations by AAALAC International [69] defined minimum standards for care and humane treatment. This was the first stage in the development of research ethics—based on protection rather than participation.

In Europe, a key document became Directive 2010/63/EU [55], which embedded the 3R principle (*Replacement, Reduction, Refinement*)—replacing, reducing numbers and refining methods [70]. In practice, these principles mean that any experiment involving animals must be scientifically justified, necessary for the development of knowledge and conducted in a way that minimises stress and suffering.

In the context of interspecies communication research, however, the 3R principle acquires a new meaning. Whereas in medical studies animals can often be replaced by computer models or cell cultures, in research on human–animal relations the subject of knowledge is precisely that relationship. This implies a need to redefine the very notion of “experiment”—from a procedure of intervention to a process that engages a pre-existing relationship of co-participation [15,46]. This shift is consistent with recent proposals to incorporate animal agency directly into research design, treating the subject’s choices, willingness, and contextual autonomy as methodological variables rather than “noise”. Incorporating agency can improve both ethical robustness and interpretive validity in behavioral and neuroscience research, especially where communicative interaction is the phenomenon under study [71].

As Haraway emphasises, the relationship between scientist and animal is dialogical: “knowing is not an act of domination, but a meeting with another” [15]. The researcher’s responsibility therefore does not end with limiting pain or stress, but also includes respect for animals’ emotionality and intentionality.

In this context, the concept of an ethics of co-participation has emerged, developed within posthumanism and relational ethology. According to Bekoff, research on animal behavior should take place under conditions that allow spontaneous behavior and emotional expression [37]. This approach is reflected in the newer guidelines of the Federation of European Laboratory Animal Science Associations (FELASA, 2020) [72], which state that studies on behavior and communication require not only humane treatment but also “recognition of animals’ cognitive potential”.

Contemporary research ethics increasingly integrates biological and philosophical standards. As de Waal writes, “empathy and morality are not a human luxury but a survival mechanism for social species” [13]. This implies that the 3R principles should be complemented by a fourth “R”—Respect—referring to animals’ subjectivity and communicative capacities.

In interspecies communication research, the idea of “cognitive respect” thus becomes especially important. Here, the animal is not an object of manipulation, but a partner in knowledge—their willingness, voluntariness and emotional comfort are crucial for both the ethical and methodological value of the study [47].

The evolution of ethical principles in science reflects a broader paradigm shift: from an ethics of protection to an ethics of relation. This shift aligns with wider posthumanist philosophy, in which humans are no longer the central subject of knowledge but part of a network of interdependent cognitive and emotional relations [16].

Recent FELASA guidelines emphasize that behavioral and communication research must account for emotional welfare and allow spontaneous expression of species-specific behaviors, integrating affective well-being into ethical evaluation [72].

### 5.3. From an Ethics of Protection to an Ethics of Relation

Traditional approaches to research ethics focused primarily on protecting animals from pain and stress. The aim was to minimise suffering rather than to understand the emotional and cognitive dimensions of human–animal relations. However, contemporary trends in bioethics, relational ethology and posthumanism call for a move away from a paternalistic model towards an ethics of relation—grounded in recognising the animal as a subject capable of experiencing emotions, making decisions and participating in cognitive processes [15,48].

A turning point for modern reflection on animal rights was Peter Singer’s *Animal Liberation*, which introduced the notion of “equal consideration of interests”. Singer argued that the capacity to suffer provides a sufficient basis for moral protection, irrespective of species membership [67]. Following him, Tom Regan [73] described animals as “subjects-of-a-life”, endowed with intrinsic value that cannot be reduced to their usefulness for humans.

At the turn of the twenty-first century, these ideas were further developed by posthumanist and feminist ethology scholars—especially Donna Haraway, Cary Wolfe and Rosi Braidotti [74]. Haraway introduced the concept of *companion species*, emphasising that knowledge and ethics are co-participatory: humans and animals co-create cognitive and emotional reality, and scientific research is a form of dialogue [15]. Wolfe describes this shift as a posthumanist ethics of co-presence, moving away from hierarchy towards relational interdependence [16].

In the same spirit, Braidotti argues that interspecies relations cannot be interpreted through the lens of domination but rather through co-existence within a shared continuum of life [74]. An ethics of relation thus becomes an ethics of co-feeling, grounded in empathy and mutual responsiveness.

In parallel, within contemporary ethology, authors such as Bekoff and de Waal demonstrate that empathy and morality are not uniquely human, but constitute a common survival mechanism for social species [13,37]. This means that emotions have not only adaptive but also ethical functions—they shape prosocial behaviors, cooperation and care for others.

In light of these concepts, research on interspecies communication cannot be conducted solely from the perspective of human cognitive goals. As Haraway stresses, an ethics of relation demands responsibility—the capacity to respond to the presence of another who is different but co-participates in the world [15]. The researcher’s responsibility therefore lies not only in protecting animals but also in recognising their active role in scientific knowledge production.

Modern research ethics is thus not merely about reducing suffering but about creating a shared space for humans and animals—a space in which knowledge, empathy and trust form the basis of co-existence. As Wolfe succinctly puts it, “knowledge without compassion is a form of moral blindness” [16].

### 5.4. The Animal as an Epistemic Participant

In research on interspecies communication, there is a growing shift in paradigm: from seeing the animal as a reacting object to recognising it as an active participant in the process of knowing. This is one of the most significant transformations in contemporary ethology and behavioral science.

In the classical research model, the animal functioned as a tool—intended to reveal laws governing human cognition. However, discoveries in the neurobiology of emotion, comparative cognition and relational ethology have shown that animals are not passive recipients of stimuli but partners in meaning-making [13,37,40].

As Sue Savage-Rumbaugh notes, in studies on bonobo chimpanzees the process of language learning does not consist of training, but of co-creating a symbolic communicative space. The animal participates in cognition, influencing its course and scope—thus becoming an epistemic subject [52]. Similar phenomena can be observed in dog cognition studies, where animals participate in experiments voluntarily and research settings are adapted to their emotional comfort [25].

Contemporary relational ethology assumes that knowledge about animals arises through dialogue, not domination. Haraway emphasises that knowing is co-participatory—an ethical event in which both human and animal are transformed [15]. In this sense, every study becomes a form of encounter rather than mere observation.

This line of thinking is also reflected in the methodology of applied behavior analysis and contemporary cognitive ethology, which aim for maximum alignment with animals’ natural behavioral repertoires. Coercion is replaced by positive reinforcement and voluntary participation, leading to more reliable data and reduced stress for the individuals studied [67].

In research on canine communication and emotion, non-invasive neuroimaging (e.g., fMRI) is employed to analyse brain responses without immobilising animals [59]. This approach not only improves welfare but also brings experimental conditions closer to real-life human–animal interactions.

As de Waal writes, “cognition is not an internal process but a dynamic relation—it arises in interaction with another subject, regardless of species” [14]. Recognising animals as co-participants in research is thus not only an ethical requirement but also a condition for the development of a science that seeks to understand consciousness in its full, cross-species dimension.

Finally, according to the principles of relational ethics and Responsible Research and Innovation (RRI), animal participation in research cannot be considered solely in welfare terms. It also includes rights to choice, withdrawal and emotional autonomy, which constitute some of the newest methodological standards in behavioral and ethological sciences [75].

As a result, the animal ceases to be an object of research—it becomes a partner in knowledge, and its experience, emotions and behavior co-create the space of scientific understanding of the world.

### 5.5. Legal Regulations and Ethical Practice

Ethics of scientific research involving animals is today not only a moral but also a legal matter. In the European Union, the primary document regulating this sphere is Directive 2010/63/EU on the protection of animals used for scientific purposes [55]. It stipulates that every experiment must be scientifically justified, carried out by competent persons and approved by an independent ethics committee.

The Directive, expanding on earlier OECD [76] and Council of Europe standards [77], obliges researchers not only to minimise suffering but also to consider animals’ cognitive and emotional capacities. Each research project therefore requires an assessment of both physiological welfare and the emotional state of the species under study. In Poland, these principles are implemented by local ethical committees for animal experimentation, operating under the Ministry of Science and Higher Education, in accordance with the Act of 15 January 2015 on the protection of animals used for scientific or educational purposes [78].

In practice, as Boissy et al. note, ethical assessment cannot be limited to physiological indicators of stress or pain [41]. Behavioral and emotional parameters are increasingly incorporated, such as facial expressions, vocalisations, affiliative behaviors and distress signals [51,67]. This perspective aligns with modern concepts of an ethics of empathy and an ethics of relation, which emphasise that emotional welfare is an integral part of research quality [15,48].

New guidelines from organisations such as FELASA (Federation of European Laboratory Animal Science Associations, 2020) [72] and the ARRIVE 2.0 Guidelines [79] stress methodological transparency, procedural validation and reporting of emotional aspects of welfare. Thus, animal welfare is no longer an ethical add-on but a key criterion of scientific quality.

In the context of interspecies communication research, the notion of “informed participation” is gaining importance. Although animals cannot formally give consent, their voluntariness and positive attitude—for example, active approach to interaction or the possibility to withdraw—constitute an ethical equivalent of consent [15,75]. This approach is applied, for instance, in modern behavioral laboratories (e.g., the “Family Dog Project” in Budapest), where dogs participate in experiments in comfortable settings, free from coercion and isolation [25].

Ethical responsibility also extends beyond the experiment itself. It includes the manner in which animals are cared for after the study, decisions about potential involvement in further projects and ensuring appropriate living conditions. As Bekoff emphasises, “the ethics of research does not end with publication of the results; it lasts as long as the interspecies relationship continues” [37].

Contemporary approaches to regulating animal research increasingly integrate law, philosophy and ethology. Within EU programmes such as Horizon Europe (Cluster 6—Biodiversity and Animal Welfare) [80], the principle of Responsible Research and Innovation (RRI) is implemented, linking scientific ethics with social practice and animals’ emotional welfare [81].

As a result, a new model of applied ethics is emerging, encompassing three levels: 1. Regulatory—compliance with legal provisions; 2. Moral—recognition of the animal as a subject capable of emotion and decision-making; 3. Cognitive—treating the human–animal relationship as co-participatory in the process of knowing (Directive 2010/63/EU [55], ARRIVE [79], FELASA [72]).

In this understanding, ethics becomes an integral part of science—not an external obligation but a component of its epistemological structure.

### 5.6. Ethical Dilemmas in Contemporary Research on Consciousness

The development of neurobiology, cognitive ethology and artificial intelligence has brought research on animal consciousness into an area that is not only scientific but also deeply ethical. Modern experiments, employing advanced neuroimaging technologies, neuronal activity recording and AI models to analyse emotion, raise questions about the limits of acceptable interference in the minds and emotions of other beings [56,82].

Studies on empathy, emotions and theory of mind increasingly use invasive techniques—implantation of electrodes, pharmacological blockade of oxytocin receptors, or experiments involving controlled sensory deprivation [45]. Although these approaches help elucidate neurobiological mechanisms of social behavior, they raise ethical concerns: Can suffering be induced in the name of studying empathy? Does researching emotion justify violating it?

As Haraway points out, the boundary between knowledge and violence is thin: “every experiment is an encounter that demands responsibility” [15]. In the spirit of her concept of *response-ability*, responsibility is not limited to limiting pain but includes recognising the other as a co-participant in knowledge.

New technologies, such as brain–computer interfaces (BCI) and AI-based systems for analysing animal emotions, open entirely new possibilities but also new domains of ethical dilemmas [49]. Machine learning models can now predict emotional states in dogs, primates or pigs based on micro-expressions and bioacoustic parameters [42]. However, interpreting such data without context may lead to oversimplifications and erroneous conclusions—algorithms may “attribute” emotions that the animal does not in fact experience. A related interpretive risk concerns anthropomorphic framing of prosocial and care-like behaviors, where narrative labels may exceed what the available evidence supports. Analyses of epimeletic behaviors caution that “rescue” or “helping” interpretations may reflect human-centered attribution rather than species-specific social ecology [83].

In this sense, technology becomes a new participant in the cognitive relationship—not a neutral tool, but a mediator of interpretation. As Wolfe notes, studying the consciousness of other beings requires an “ethics of interpretation” that acknowledges the limits of human knowledge and refrains from claiming total transparency of animal minds [16].

Donaldson and Kymlicka adopt a similar position in their concept of *Zoopolis*, according to which animals should be treated as moral co-citizens of a shared cognitive community [17]. This implies that any research involving animals should respect principles of voluntariness and avoid situations in which the cognitive relationship turns into domination.

Ethical challenges also arise in comparative studies of consciousness—such as mirror tests or experiments on recognising intentions and emotions. As de Waal (2022) notes, the issue is not whether animals are “conscious like humans”, but that humans attempt to evaluate their consciousness solely by human standards [14]. Such an anthropocentric model of knowledge reinforces species hierarchy and ignores the specificity of non-human forms of mind.

From the standpoint of relational and posthumanist ethics, the key challenge for contemporary science is therefore to develop methods that make it possible to study consciousness without violating it. Instead of intervening in neurophysiology, increasing emphasis is placed on participant observation, field ethology and non-invasive technologies such as fMRI, vocal, gestural and facial analysis [49,51,59].

As Bekoff underscores, “understanding the emotions and consciousness of other species does not require domination but empathetic co-existence” [37]. In this spirit, posthumanist ethics of knowledge combines philosophical reflection with scientific responsibility—redefining the experiment as dialogue rather than intervention.

### 5.7. From Laboratory to Participatory Research

Alongside the development of ethics in animal research, a new model of science is emerging—participatory science, in which animals and their guardians co-create the process of knowing. This direction has arisen as a response to the limitations of the classical laboratory model based on control, isolation and experimentation [84].

In traditional laboratories, an instrumental approach prevailed—the animal was a “model” used to test hypotheses about humans. Contemporary approaches, inspired by an ethics of relation and co-participation, assume that research is dialogical [14,15,16,37]. Knowledge does not arise through domination but through cooperation and trust.

One pioneering example of this approach involves projects that engage dog guardians in observing their animals’ behavior and emotions in home environments [84]. In this way, data on emotions and communication are not distorted by laboratory stress, and findings better reflect natural human–animal relationships.

In canine research, methods based on voluntary participation and positive emotional states are increasingly used, such as “dog-friendly fMRI”, reward-based training or cognitive tests conducted at home [25,59]. Rather than forcing the animal to perform tasks, the researcher observes spontaneous behavior and analyses communication in its natural context.

From a philosophical perspective, the participatory model aligns with posthumanist epistemologies of relation, according to which knowledge is co-created by multiple subjects—human and non-human [16,74]. In this framework, experiments become cognitive encounters rather than tests. As Braidotti notes, “contemporary science requires not only intelligence but empathy—the capacity to co-feel with the object of knowledge” [74].

In practical research, implementing participatory science entails

1. Replacing invasive studies with observation in natural environments;
2. Involving animal guardians and local communities in data collection;
3. Moving research from laboratories into everyday life settings (homes, training centres, reserves);
4. Recognising the animal as a co-creator of knowledge—an active participant in the cognitive relationship, not merely a data source [84].

This model combines scientific rigour with empathy and trust, resulting in a better understanding of the emotional and social dimensions of communication. Moreover, it increases public acceptance of research, as it respects animals’ moral status and welfare.

As de Waal emphasises, “we cannot understand animals if we do not allow them to speak with their own voices” [13]. Participatory science makes this possible—giving voice to those who have until now been mere objects of observation, and creating a shared space of knowledge.

### 5.8. The Philosophical Dimension of Ethics in Communication Research

Ethics of research on interspecies communication is not limited to procedural rules or legal regulations. It primarily concerns how humans understand knowledge, consciousness and relationships with other beings. Its philosophical dimension indicates that the researcher–animal relationship is not merely a technical interaction but an encounter between two cognitive worlds—human and non-human [85].

Contemporary philosophical currents, particularly posthumanism and relational bioethics, redefine the concept of subjectivity in science. According to Wolfe, research on animal consciousness and language requires a “posthumanist epistemic shift”—moving from domination-based knowledge to co-participatory knowledge [16]. This means that the animal is no longer an “object” but a participant in a shared process of meaning-making.

Donna Haraway advances a similar position, introducing the notion of *response-ability*—the capacity to respond to the presence of another. Responsibility in research does not consist in controlling, but in listening and responding. It is an ethics of attentiveness in which the researcher recognises that every cognitive relationship entails a moral commitment to the other being [15].

In this context, the scientific experiment becomes an ethical practice—a form of co-creating knowledge rather than a one-sided act of observation. As Braidotti argues, knowledge requires the “transgression of anthropocentrism”—overcoming purely human perspectives in favour of a multi-species cognitive horizon [74].

Philosophical reflection on interspecies communication also draws on phenomenological accounts of relation—from Merleau-Ponty [86] to contemporary theories of embodied cognition [87]. In this view, cognition is not abstract but embodied and situated within interactions. Humans perceive the world through bodies and emotions—just as animals do. This shared sensory experience forms the basis of empathy and understanding.

From this perspective, ethics of communication research is not merely a set of rules but a practice of co-presence. As Bekoff notes, “morality is a way of life, not a code” [37]. An ethics of relation thus consists of co-feeling and in acknowledging that knowledge emerges from shared experience.

It is noteworthy that philosophical reflection on interspecies relations increasingly converges with the natural sciences. De Waal argues that empathy and morality are not cultural constructs but biological mechanisms of cooperation [14]. Philosophy and biology thus meet at a point where knowledge becomes shared, and interspecies boundaries grow fluid.

Consequently, the philosophical dimension of ethics in communication research leads to a new form of epistemology—an epistemology of relation. At its centre is the conviction that cognition is dialogical and that ethics and science are inseparably linked. Knowledge without empathy is incomplete, and empathy without reflection is powerless.

In this sense, an ethics of knowledge becomes the foundation of future science: a science that not only describes the world but co-creates it—together with other species.

### 5.9. Summary

Ethics of research on interspecies communication is evolving from protecting animals from suffering towards recognising them as active participants in knowledge. Contemporary approaches, integrating posthumanist philosophy, bioethics, ethology and cognitive science, indicate that cognition is not one-sided but co-created by different species and forms of consciousness [14,15,16].

Traditional experimental ethics—focused on minimising pain and stress—is giving way to an ethics of relation, in which co-participation becomes the central value. The researcher not only observes but collaborates, and the animal is no longer an “object” but a partner in meaning-making. This has not only moral but also methodological consequences—it improves the reliability of studies, reduces stress and strengthens the authenticity of interactions [25,37,81].

As Haraway emphasises, genuine responsibility in science is *response-ability*—the capacity to respond to the presence of another being, regardless of species [15]. This responsibility cannot be reduced to fulfilling formal ethical criteria; it requires attentiveness, co-feeling and empathetic understanding.

In this context, contemporary research ethics expands its scope:

1. **Ontologically**—by recognising the multi-species nature of cognition;
2. **Epistemologically**—by treating communication as a shared discovery of meanings;
3. **Axiologically**—by attributing value to emotions, empathy and relationality as components of knowledge [14,74].

The new ethics of communication research thus demands a redefinition of scientificity. Knowledge is no longer an act of dominance over the world but a process of cognitive co-participation, in which humans share responsibility for a common experience of existence with other species [14,37].

As de Waal notes, understanding emotions and morality in animals teaches us humility about our own nature: “Empathy is not a luxury of humans—it is a condition of survival for social species” [14]. This insight sets the direction for the development of modern research ethics—empathetic, dialogical and co-participatory.

Ultimately, philosophical and ethological reflection on communication leads to a new definition of knowledge: knowledge grounded in relation rather than domination. Ethics and epistemology here form a single structure—a science that is responsible, empathetic and co-created by all beings capable of emotion and relation.

## 6. Interdisciplinary Discussion: From Biology to Posthumanist Philosophy

### 6.1. Introduction—From Communication to Co-Participation

Contemporary research on interspecies communication has shifted its focus from asking whether animals can communicate to examining **how**, and in what sense, they co-create meaning together with humans. Communication is no longer seen as a one-way transfer of information but as a process of co-participation, in which emotions, empathy, and intentions shape a shared cognitive space [14,15,37].

An interdisciplinary approach brings together neurobiology, ethology, cognitive science, philosophy of mind, bioethics, and AI studies. Each of these fields offers its own language of description, yet all converge on a single core problem: the relational nature of cognition. Only their integration makes it possible to grasp the complexity of phenomena in which communication, emotion, and consciousness intersect on multiple levels [85,86,87].

In this perspective, animals cease to be mere “models” and become partners in the process of discovering the world. Biology provides descriptive tools, philosophy offers ethical reflection, and technology opens new possibilities for analysing communication. This interdependence of disciplines points to the emergence of a new *epistemology of relation*, in which knowledge is no longer an act of domination but a practice of co-creating meaning [16,74].

### 6.2. Biological Foundations of Relationality

From a biological standpoint, communication and empathy share common neurobiological foundations. The activation of mirror neurons, the release of oxytocin and dopamine in social interactions, and the involvement of the cingulate cortex and insula all indicate that co-feeling is a biological, not merely cultural, phenomenon [13,39,59]. Convergent evidence links affective appraisal and interoceptive processing to insular and cingulate networks coordinated with amygdala-centered salience systems across mammals, supporting the view that social–affective coupling relies on conserved neural architecture. Neurocognitive evidence indicates that affective processing relies on deeply conserved subcortical and interoceptive circuits, including the insular cortex, which integrates bodily feedback with emotional appraisal across mammalian species [88].

The discovery of mirror neurons in macaques [62], and the identification of analogous mechanisms in humans and dogs [59], suggest that the capacity to recognize the emotions of others evolved as an adaptive mechanism [8,13,59]. Empathy and cooperation are therefore not exceptions but *survival strategies* in social species [13,37].

As Bekoff argues, emotions and morality have a shared origin in the biology of relations: morality is not a human luxury but a condition for living together in a community [37]. Biology thus reveals that communication is not simply a tool for information transfer but a component of a social system for regulating emotions.

In this sense, relationality is phylogenetic: it emerges wherever cooperation yields greater adaptive benefits than competition. In humans, dogs, and primates, this means the ability not only to respond to others’ emotions but also to create shared meanings through affective resonance [13,25].

### 6.3. Embodied Cognition

Embodied cognition emphasises that perceptual and motor systems structure cognitive processes. In animals, meaning arises through bodily engagement with the environment rather than abstract symbolic manipulation.

Interdisciplinary findings integrating cognitive science, neurophenomenology, and ecological psychology support this view. Theoretical models developed by Merleau-Ponty, Varela, Thompson, and Rosch show that cognition emerges from dynamic coupling between organism and environment [86,87]. Applications in comparative ethology demonstrate that animals coordinate bodily rhythms, gaze patterns, and micro-movements during interactions, creating shared affective fields in which meaning is co-constructed rather than transmitted [37,89].

### 6.4. Technology as a New Interspecies Language

AI, bioacoustics, and machine vision technologies allow researchers to decode affective and semantic patterns in animal communication, extending human perceptual capacities. Large-scale models trained on raw audio—such as *animal2vec* and MeerKAT—reveal structure in rare-event vocalisations and enable clustering of emotional states across species [90]. Multimodal systems such as NatureLM-Audio integrate acoustic and linguistic embeddings to detect contextual meaning in complex communication systems [34]. Projects like the Earth Species Project demonstrate how foundation models can function as a mediating technological “language”, translating statistical regularities into interpretable communicative patterns [12].

These tools do not replace ethological observation; instead, they supplement human analysis by identifying acoustic, visual, and behavioral features that would otherwise remain inaccessible. AI-based classifications should be validated against ethological observation, physiological indicators, and expert behavioral annotation. Multimodal convergence and transparent benchmarking are essential to prevent overinterpretation. In welfare science, AI-assisted assessment is increasingly used to monitor stress, positive affect, and synchronised behavior in domesticated species [56,82]. Yet algorithmic precision introduces interpretive risks: emotional states may be reduced to computational categories unless contextualised within species-specific emotional ecology.

Ethical frameworks—including ARRIVE 2.0, FELASA, and Responsible Research and Innovation—emphasise that technological development must remain grounded in ethical transparency and contextual judgment [56,68,72,79,81]. These guidelines underline that AI should extend, not replace, ethological understanding, and that algorithmic interpretations must avoid anthropocentric bias and oversimplification of animal experience [90,91]. Technology can serve as a new interspecies language only when embedded within an ethical and relational framework that recognises animals as partners in meaning-making.

This ethical–epistemic model marks a new perspective in human–animal relations. Moving away from hierarchical knowledge towards co-participation allows a deeper understanding of the emotional dimension of communication and helps avoid technological simplifications. Rather than “translating” animal speech, we should learn to *listen*—in both an epistemic and ethical sense.

### 6.5. The Limits of Language and the Possibility of Understanding

The limits of language have long been central to philosophical reflection. Wittgenstein famously stated that the limits of one’s language are the limits of one’s world, yet contemporary research on interspecies communication shows that the world of meaning extends far beyond words [92]. A dog responding to tone of voice, a chimpanzee using gesture, or a dolphin reproducing sound sequences all demonstrate that understanding does not require a shared lexical system but a shared *emotional intentionality*.

From a biological standpoint, interspecies communication is grounded in shared emotions and intentions, as confirmed by research on mirror neurons, empathy mechanisms, and theory of mind in primates and dogs [8,13,14,32]. Consciousness thus appears as a relational phenomenon: it emerges where two organisms respond to each other in the rhythm of emotion and action. Language becomes one medium among many—not the sole condition of understanding.

In parallel with theoretical reflection, empirical work has advanced. From the symbolic communication experiments with primates in the 1960s to contemporary AI systems that analyse vocalisations, micro-expressions, and physiological signals, science has moved from a behavioral paradigm to a holistic model of affective cognition. The main stages of this development are summarised in Table 3.

This progression highlights that interspecies understanding is a dynamic process: it moves from simple conditioned responses to conscious exchange of emotions and intentions. At the same time, technological development raises questions about the limits of interpretation. Can AI truly “understand” animal emotions, or does it merely classify them?

As de Waal reminds us, empathy is not calculation but co-participation [14]. For this reason, each technological advance must be embedded in ethical reflection, which insists that communication is not just signal exchange but shared meaning-making. The limits of language thus become the limits of *sensitivity*: where science loses empathy, it also loses understanding.

Neurocognitive and comparative studies indicate that the ability to interpret intentions and emotional states precedes the evolution of complex linguistic skills, supporting the view that the foundations of communication are pre-linguistic and affective [8,37,40].

### 6.6. Interdisciplinarity as a New Paradigm

Research on interspecies communication increasingly requires crossing disciplinary boundaries. Biology, philosophy, neurocognitive science, ethology, and AI studies now form a common field in which emotion, consciousness, and language are analysed in an integrated manner. As Bekoff notes, morality and communication are not uniquely human attributes but part of the shared evolutionary heritage of social species [37]. Interdisciplinarity is therefore not a methodological option but a necessity dictated by the nature of the subject itself: relations between sentient beings.

Biologically, communication is a neurobiological and affective process based on resonance, oxytocin coupling, and mirror neuron activation [10,14,37]. For ethologists, it is an adaptive system that supports group survival and cooperation [2,13]. For philosophy and ethics, it is a moral space in which reciprocity and shared responsibility emerge [15,16,17]. Integrating these perspectives reveals that understanding across species is not merely a matter of signal interpretation but a form of joint participation in a shared world of meaning.

Interdisciplinarity requires not only collaboration across fields but also a shift in epistemic paradigm. Haraway’s concept of *situated knowledge* emphasises that cognition always depends on context, body, and relation. Bringing together biologists, ethologists, philosophers, psychologists, sociologists, and AI specialists enables a more complete picture of human–animal relations.

In practice, laboratories increasingly combine neuroimaging with behavioral analysis, while applied ethology projects integrate physiological data with emotional assessments. The use of AI does not replace empathy with algorithms but rather builds a bridge between data-driven methods and ethical reflection. The emergent approach—sometimes described as *affective data science*—combines quantitative data (biometrics, acoustics, movement analysis) with qualitative interpretation of behavior and emotional context.

As de Waal argues, understanding animals requires not scientific distance but empathic closeness [14]. Interdisciplinarity thus becomes an ethical stance: a willingness to learn from other forms of life. The biologist must understand ethical theory; the humanist must understand the neurobiology of emotion. Only then can we fully grasp processes in which emotion, consciousness, and technology form a single, interconnected cognitive system.

In this light, interdisciplinarity is not simply joint authorship but a new paradigm of science—one that links empathy with rigour and data with lived experience. It enables the creation of knowledge that not only describes but also respects a world in which humans are just one of many participants in communication. The conclusions of such research naturally lead toward a broader ethical reflection: if communication and cognition are relational, science itself must become an act of co-participation. This is where the ideal of *ethics of co-participation* emerges, as the logical extension of the interdisciplinary paradigm and the foundation of a new relational humanism.

### 6.7. Towards an Ethics of Co-Participation

The accumulated evidence from interspecies communication research shows that the relationship between humans and animals can no longer be described in terms of domination or instrumental use. Contemporary knowledge from biology, ethology, philosophy, and cognitive science points to the relational character of both cognition and morality: they arise in the space of encounter, not hierarchy [13,15,16,17]. Communication becomes less a matter of information transfer and more a process of emotional and cognitive co-participation, in which meanings are co-created by sentient beings.

An ethics of co-participation, inspired by Haraway and Wolfe, assumes that humans are not detached observers but participants in a multispecies web of life [15,16]. Ethical responsibility (*response-ability*) does not consist solely in protecting animals from humans but in recognising them as subjects who co-constitute knowledge. Every experiment, interaction, or observation is thus a form of dialogue—and every scientific decision becomes a moral act.

In biology and clinical ethology, animal welfare is increasingly understood as encompassing emotional and cognitive domains, not just physical health [10,14,41]. Incorporating an ethics of co-participation into research practice means moving from control-based models to relational ones—from experiment to partnership. This requires a different approach to study design: the animal is no longer a “measurement object” but a communicative partner whose reactions, preferences, and emotions shape the research process itself.

Philosophically, this ethics aligns with relational posthumanism, which redefines humanism. Rather than placing humans at the centre, it emphasises their interdependence with other forms of life and with technological agents. As Braidotti argues, the future of ethics lies in *expanding the field of care* to include animals, machines, ecosystems, and non-verbal organisms [74]. In this context, interspecies communication is not only a topic of research but also a moral practice: a way of cultivating a new sensitivity toward the world.

An ethics of co-participation does not abandon scientific rigour; it extends it to include empathy and responsibility. It demands of the researcher not only technical competence but also the ability to listen, to observe, and to interpret emotions within their biological and social context. As de Waal stresses, morality is not a human invention but a biological mechanism that sustains cooperation in social species [13,14]. Ethics has its roots in emotion, and cognition in relation.

For this reason, future science should develop in the spirit of *affective cognition*—understanding based on co-feeling rather than distance. Emotions, empathy, and relationality form the foundation of a new epistemological model, in which knowledge and morality are inseparable.

Many disciplines are already moving in this direction—from ethology and neurobiology to bioethics, philosophy of technology, and AI studies. They share the conviction that understanding the world requires participation, not domination. This implies a shift from *science about animals* to *science with animals*: research grounded in mutual respect, empathy, and shared responsibility for coexistence. In this sense, an ethics of co-participation forms the foundation of a new epistemology of relation, developed further in the final conclusions of the article.

### 6.8. Synthesis of the Discussion

The analysis of interspecies communication from biological, ethological, and philosophical perspectives indicates that this phenomenon is not merely a process of transmitting information, but a fundamental form of shared cognitive and emotional participation.

The evolutionary continuity of mechanisms underlying emotion, empathy, and intentionality demonstrates that interspecies boundaries are functional rather than ontological [8,10,14,40]. Shared biological foundations—such as the limbic system, oxytocin-mediated feedback loops, and mirror neuron activation—enable forms of affective resonance and mutual emotional alignment.

**Figure 4:**
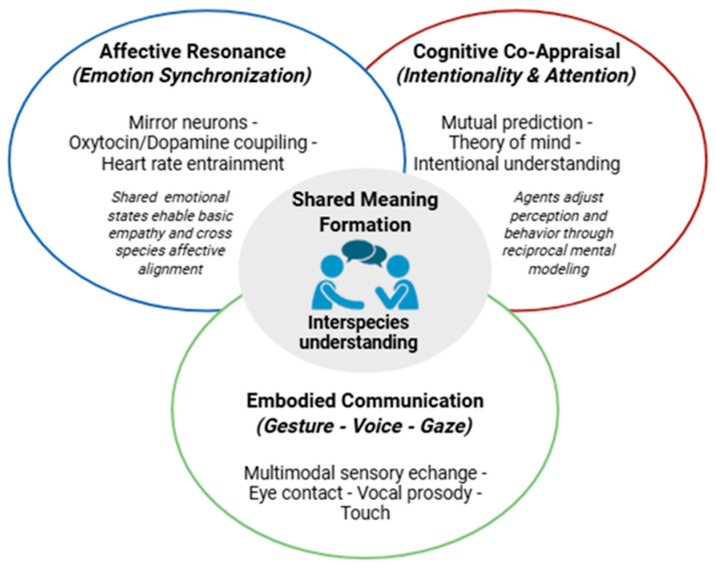
**Conceptual model of interspecies meaning formation.** Figure 4 illustrates a triadic model of shared meaning creation integrating: (1) Affective Resonance—emotional synchronisation and hormonal coupling (oxytocin, dopamine); (2) Embodied Communication—multimodal exchange via gesture, voice, and gaze; (3) Cognitive Co-Appraisal—mutual adjustment of intentions and attention. Understanding arises not from linguistic symmetry but from affective alignment and embodied intentionality, enabling interspecies empathy and co-created meaning [10,14,15,16,37,40].

Contemporary interdisciplinary research shows that interspecies communication constitutes a complex system integrating three levels of cognition: affective, embodied, and cognitive. The affective level includes emotional resonance and cross-species physiological coupling (e.g., human–dog). The embodied level is expressed through gesture, voice, posture, and gaze. The cognitive level involves the recognition of intentions and the sharing of attention. This three-dimensional structure, represented earlier in the conceptual model (Figure 4), provides the foundation for a shared “language of emotion and meaning”.

At the same time, technological developments in bioacoustics, machine vision, and artificial intelligence introduce new possibilities for the study of communication. AI models based on multimodal data (vocalization, movement, physiology) are increasingly capable of detecting emotional states in animals with high precision [36,90].

However, technological progress without ethical reflection risks reducing complex emotional relations to statistical patterns. For this reason, integrating technology with ethics becomes essential—particularly within the posthumanist framework of responsibility for interpreting the experiences of other beings [15,16,17]. A recurring theme throughout the previous sections is the relational nature of cognition—the idea that understanding arises through interaction rather than unilateral observation.

In both primates and dogs, emotions and empathy are not only biological mechanisms, but also the foundations of cooperation, moral behavior, and cultural transmission [9,10,14,19].

Communication—whether verbal, gestural, acoustic, or technologically mediated—thus functions as a cognitive bridge that integrates mind, body, and emotion into a shared system of meaning.

From a philosophical standpoint, these findings lead to a redefinition of humanism. The human being is no longer the center of cognition but a participant within a network of relations that includes other species and emerging technologies.

In this view, interspecies communication becomes a metaphor for a new relational epistemology in which knowledge and ethics are inseparable. As de Waal notes, **“empathy is not a moral luxury but a biological condition for the survival of social species”** [14]. Future science, if it is to remain responsible, must integrate knowledge with care, precision with attentiveness, and technology with empathy.

The conclusions drawn from this discussion indicate that the future of interspecies communication research requires an integrated approach combining empirical evidence with ethical and philosophical reflection.

It is precisely this unity of biology, technology, and ethics that sets the direction for modern science—toward an epistemology of participation explored further in the next section.

This interpretation aligns with embodied cognition frameworks formulated by Varela, Thompson and Rosch [87], and later developed within cognitive science by Clark [89], emphasizing that understanding emerges through dynamic embodied interaction rather than symbolic decoding. Recent technological advances—ranging from multimodal acoustic analysis [36], self-supervised bioacoustic transformers [90], and semantic mapping models such as NatureLM-Audio [34] to domain-specific systems for decoding emotional structure in vocalizations [91]—highlight both the potential and limitations of AI-based interpretations of non-human communication. These developments resonate with posthumanist perspectives, including Braidotti’s relational ethics [74] and Rowlands’ defence of non-human moral agency [85], both of which frame communication as a shared epistemic domain rather than a human-centred hierarchy.

### 6.9. Summary of the Discussion

The synthesis of biological, cognitive, and technological findings shows that communication is an integrated affective–cognitive process shaped by evolutionary, ecological, and social factors. Shared emotional mechanisms, multimodal perception, and context-sensitive interaction underpin communicative behavior across species.

Recent contributions from affective neuroscience, welfare science, and posthumanist ethics argue that emotions are central to communication and cooperation [41,42,45,74]. Complementary AI-based analyses reveal that multimodal emotional signals can be identified, clustered, and mapped with increasing precision, enhancing our ability to document cross-species interaction [34,36,56,82]. Together, these perspectives support a relational model of cognition in which meaning emerges through shared emotional engagement and ethically grounded interpretation.

## 7. Conclusions—Toward a New Epistemology of Relation

Advances in biological, neurocognitive, and ethological sciences have profoundly transformed our understanding of animals and their place within the broader landscape of cognition. Discoveries concerning mirror neurons, oxytocin-mediated emotional coupling, and the capacity of animals for empathy demonstrate that communication, morality, and affective attunement are not exclusively human traits [10,14,26,40]. Interspecies boundaries appear to be far more cultural than biological, and consciousness itself emerges as a **relational phenomenon**, co-created within the space of interaction.

From a biological perspective, communication functions as an adaptive mechanism enabling cooperation and the survival of social species. From a philosophical viewpoint, it constitutes a form of participation in a shared world of meanings, in which knowledge and ethics are inseparably intertwined. From an ethical standpoint, communication becomes a domain of responsibility in which every interspecies encounter requires attentiveness and respect for difference [13,51]

A **new epistemology of relation** redefines the very notion of knowledge. Understanding is no longer a unilateral act of observation but a process of co-creating meaning among sentient beings. Humans, animals, and technologies form a network of shared participation in which emotions, gestures, vocalisations, and algorithms converge into a common language of empathy and coexistence. As Haraway observes, “learning with other species is learning responsibility” [15]—and this shift from observation to participation constitutes the core of future scientific inquiry.

The recognition of animal emotions and consciousness leads toward a new form of humanism—**relational humanism**—in which humans coexist with other life forms within a shared sphere of meaning. Contemporary ethology, neurobiology, and philosophy show that language is not merely an instrument of communication but a **mode of being-in-the-world**. Every canine gaze, chimpanzee gesture, or dolphin vocalisation becomes an invitation to dialogue—an indication that knowledge and compassion are fundamentally unified practices.

Thus, the science of the future must integrate empiricism with empathy, intelligence with care, and technology with ethics. The new epistemology of relation—grounded in active participation—is not only a philosophical proposition but a **moral project for contemporary science**: a framework in which understanding is not an act of domination but a form of coexistence within a multispecies community of meaning.

This perspective is strongly supported by affective neuroscience, particularly Panksepp’s evolutionary model of primary emotional systems [40], which reinforces the idea that affective processes predate linguistic capacities and underlie all meaningful social communication. Contemporary research on animal sentience [42] demonstrates that emotion, motivation, and cognition form an integrated system that structures interspecies understanding. These findings provide renewed significance to rights-based arguments articulated by Regan [73] and relational moral theories proposed by Rowlands [85], which ground moral considerability in lived experience, vulnerability, and the emotional agency of non-human beings.

## 8. Practical Implications and Interdisciplinary Applications

Research on interspecies communication carries not only theoretical but also practical and ethical significance. Its interdisciplinary nature requires close collaboration between biologists, ethologists, philosophers, AI specialists, veterinarians, welfare scientists, and behavioral practitioners. The implications derived from this field highlight the necessity of an integrated approach in which biological knowledge is combined with ethical and social reflection.

### 8.1. Biological Sciences

Interspecies communication research requires the integration of neurobiology, ethology, cognitive psychology, and welfare science. Understanding animal emotions and empathy enables the development of cooperation models grounded in shared biological mechanisms [8,9,10]. Citizen science approaches [84] and integrative welfare AI projects [56,82] illustrate how public participation and advanced analytical tools can jointly expand the empirical basis for interpreting cross-species communication. Findings from comparative neuroethology and emotional regulation further demonstrate that communication must be interpreted through multimodal and relational frameworks rather than through isolated signals [19,22,39,40].

### 8.2. Humanities and Social Sciences

There is a pressing need to redefine concepts such as subjectivity, consciousness, and moral agency. Posthumanist philosophy, bioethics, and the sociology of animals provide conceptual frameworks for developing new research, educational, and cultural practices [15,16,17]. Posthumanist analyses by Braidotti [74] and Wolfe [16] offer guidance for incorporating relational ethics into policy design, public education, and communication strategies. These perspectives emphasise that animals must be understood as active contributors to shared meaning-making rather than passive objects of human inquiry.

### 8.3. Veterinary and Behavioral Practice

Emotional communication is increasingly recognised as central to diagnostics, behavioral assessment, and therapeutic interventions in veterinary and clinical ethology contexts. Practitioners must integrate neurobiological insights with an empathetic approach that acknowledges the animal as a communicative partner [14,41]. Phenomenological models of embodied perception [86] reinforce welfare-oriented practices that prioritise the animal’s lived emotional experience, particularly in areas such as stress assessment, cooperative handling, and behavioral rehabilitation.

### 8.4. Ethics of Scientific Research

Experiments involving animals must adhere to principles of informed participation, respect for emotional well-being, and minimisation of distress. Participatory models and non-invasive methodologies should become the normative standard rather than the exception [26,51]. International frameworks—including ARRIVE 2.0 [79], FELASA guidelines [72], OECD recommendations [76], and the European Convention for the Protection of Vertebrate Animals [77]—provide essential guidance for ensuring that emerging emotional-based approaches remain transparent, ethically robust, and aligned with global welfare standards. Ethical analyses by Regan [73], Rowlands [85], and Rollin [46] further emphasise the moral relevance of emotional agency and vulnerability in research design.

### 8.5. Technological Development (AI, Bioacoustics, Machine Vision)

AI, bioacoustics, and machine vision technologies can significantly enhance human–animal communication by enabling fine-grained detection of affective and semantic patterns. Models such as animal2vec, NatureLM-Audio, and transformer-based rare-event detectors allow the analysis of emotional states and contextualised behavior previously inaccessible to human observers [34,90,91]. However, technological innovation must be paired with ethical oversight. Algorithms should support the understanding of animal emotions rather than their instrumentalisation or reduction to abstract computational categories [41,82]. Responsible Research and Innovation frameworks [81] and welfare AI analyses [56] stress the importance of grounding technological interpretations in species-specific emotional ecology.

### 8.6. Education and Society

A new relational awareness is required for contemporary education and public engagement. Educational programmes should present animals as sentient, communicative beings capable of co-creating meaning. Insights from cognitive ethology [37], sentience studies [42], and emotional neuroscience [40] provide foundations for transforming curricula, shaping public communication, and promoting ethically informed attitudes toward interspecies coexistence. Such education forms the basis for an ethically sustainable future in a multispecies world.

A relational framework can inform welfare practices by prioritizing cooperative interaction, guide conservation monitoring by contextualizing communicative change, and support policy frameworks that explicitly recognize affective experience as ethically relevant.

## Figures and Tables

**Table 1:** **Major milestones in experimental and technological approaches to interspecies language research (1931–2025).** The table summarizes methodological and conceptual advances in interspecies communication research, showing the evolution from behavioral paradigms to AI-driven approaches capable of recognizing affective and semantic structures across species.

Year/Period	Species/Model	Lead Researcher/Institution	Key Findings/Technological Innovations	Cognitive and Ethical Significance
1931	Chimpanzee Gua (*Pan troglodytes*)	Winthrop & Luella Kellogg	Cross-fostering experiment with a human child; comprehension of human words but lack of vocal articulation.	Early evidence of semantic understanding without speech; distinction between cognition and anatomy.
1969	Chimpanzee Washoe (*Pan troglodytes*)	Allen & Beatrix Gardner, Univ. of Nevada	Acquisition of over 150 ASL signs; spontaneous sign transfer between chimpanzees.	Demonstrated symbolic learning and cultural transmission; challenged human exclusivity in language.
1971	Chimpanzee Sarah	David Premack	Use of plastic symbols to represent logical relations (color, size, order).	Evidence for abstract reasoning and symbolic categorization in non-human minds.
1979	Nim Chimpsky	Herbert Terrace, Columbia Univ.	Taught sign language; responses mainly imitative rather than generative.	Reframed debate on intentionality and true linguistic competence.
1970	Chimpanzees & Orangutans	Gordon Gallup	Mirror self-recognition test demonstrating self-awareness.	First empirical indicator of self-consciousness; foundation for theory of mind studies.
1971–1990	Gorilla Koko (*Gorilla gorilla*)	Francine Patterson, Stanford Univ.	Over 1000 ASL signs; emotional expression including humor, grief, and affection.	Showed affective depth and empathy; shifted ethics toward viewing animals as communicative subjects.
1978–1994	Orangutan Chantek (*Pongo pygmaeus*)	Lyn Miles	Use of ASL for requests, emotions, and self-reference.	Confirmed identity-based and relational communication; emphasis on individuality.
1980–2000	Bonobo Kanzi (*Pan paniscus*)	Sue Savage-Rumbaugh	Spontaneous acquisition of lexigrams and comprehension of spoken English.	Introduced relational ethology; communication as shared emotional experience.
2016–2022	Bonobos, Chimpanzees, Capuchins	Frans de Waal	Empathy, fairness, and moral emotions observed in group behavior.	Revealed emotional foundations of morality; ethics of co-experiencing rather than observing.
2023	Earth Species Project (multi-species: dolphins, birds, whales)	Earth Species Project (non-profit initiative)	Large Language Models (LLMs) applied to bioacoustic data; pattern translation and contextual meaning detection.	Promotes non-anthropocentric decoding; emphasizes preservation of species-specific semantics.
2024	animal2vec/MeerKAT	Schäfer-Zimmermann et al.	Self-supervised Transformer for rare-event acoustic signals; large-scale reference bioacoustic dataset.	New paradigm in multimodal learning; raises questions of data ownership and consent in animal datasets.
2024	NatureLM-Audio	Robinson et al., Google DeepMind	Foundation Audio–Language Model mapping animal vocalizations to semantic embeddings.	Moves toward ‘algorithmic empathy’; limits of AI interpretation of non-human meaning.
2024	Dog Bark Decoding	Abzaliev et al., University of Michigan	Human NLP models adapted for canine vocal analysis and emotion mapping.	Highlights anthropomorphic bias and need for contextual emotional interpretation.
2025	AI for One Welfare/Ethical AI Integration	Foris et al., Front. Vet. Sci. [56]	Development of welfare-oriented AI systems integrating multimodal animal data (bioacoustics, motion, vision).	Represents convergence of cognitive science, welfare ethics, and responsible AI; fosters participatory interspecies cognition.

**Table 3:** **Major milestones in experimental and technological approaches to interspecies language research (2023–2024**). The table summarises methodological and conceptual advances in interspecies communication research, showing the evolution from behavioral paradigms to AI-driven approaches capable of recognising affective and semantic structures across species.

System/Model	Type of Signal Analyzed	Core Technology	Primary Objective	Ethical Considerations	Source
**Earth Species Project (2023)**	Bioacoustic (dolphins, birds, whales)	Large Language Models (LLM)	Pattern translation and contextual meaning detection	Avoiding anthropocentric bias; preserving species-specific semantics	[12]
**animal2vec/MeerKAT (2024)**	Raw acoustic data (multi-species)	Self-supervised Transformer	Emotion and behavior classification from rare-event signals	Data ownership, transparency, dataset consent	[90]
**NatureLM-Audio (2024)**	Audio + language embeddings	Foundation Audio–Language Model	Semantic mapping of vocalizations	Algorithmic empathy and interpretive limits	[34]
**Dog Bark Decoding (2024)**	Canine vocalizations	Speech recognition via human NLP models	Bark classification and emotion mapping	Anthropomorphic misinterpretation	[91]

## Data Availability

No new data were created or analyzed in this study. Data sharing is not applicable to this article.
